# A critical size volumetric muscle loss model in mouse masseter with impaired mastication on nutrition

**DOI:** 10.1111/cpr.13610

**Published:** 2024-02-14

**Authors:** Ning Zhao, Yixuan Huang, Xu Cheng, Li Xie, Wenlin Xiao, Bing Shi, Jingtao Li

**Affiliations:** ^1^ State Key Laboratory of Oral Diseases, National Center for Stomatology, National Clinical Research Center for Oral Diseases Sichuan University Chengdu China; ^2^ Department of Oral and Maxillofacial Surgery, West China Hospital of Stomatology Sichuan University Chengdu China; ^3^ Department of Stomatology The Affiliated Hospital of Qingdao University Qingdao China

## Abstract

Orofacial muscle defect due to congenital anomalies, tumour ablation or traumatic accident that exceeds endogenous regeneration capacity may lead to sustained deficits in masticatory function and nutrition intake. Functional recovery has always been the goal of muscle tissue repair, but currently, there is no suitable model for quantitative analyses of either functional consequences or treatment efficacy of orofacial muscle defect. This study proposed a critical size volumetric muscle loss (VML) model in mouse masseter with impaired mastication on nutrition. Full‐thickness VML defects in diameter of 1.0, 1.5, 2.0 and 3.0 mm were generated in the centre of the mouse masseter using a biopsy punch to determine the critical size for functional impairment. In the VML region, myogenesis was dampened but fibrogenesis was activated, as long with a reduction in the density of the neuromuscular junction and an increase in vascular density. Accordingly, persistent fibrosis was observed in the centre region of VML in all diameters. The 2.0 mm diameter was the critical threshold to masticatory function impairment after VML in the masseter. VML of 3.0 mm diameter led to a significant impact on nutrition intake and body weight gain. Autologous muscle graft effectively relieved the fibrosis and functional deficit after VML injury in the masseter. This model serves as a reliable tool in studying functional recovery strategies for orofacial muscle defects.

## Graphical abstract

In this study, we presented for the first time an injury model in the orofacial masticatory muscle with function impairment and systemic impact, along with comprehensive quantitative histological analysis, muscle repair effector cell proliferative activity and masticatory function analysis, providing a powerful tool for testing and developing tissue engineering and regenerative medicine approaches for orofacial muscle repair.

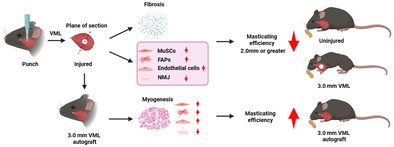

## 1 INTRODUCTION

Skeletal muscle is the most abundant tissue in the human body, comprising around 40% of our overall mass.^1^ The contraction of skeletal muscle drives body movements via their skeletal attachments, meeting a broad range of functional demands from posture maintenance, breathing, thermogenesis to body movement and more.^2^

In situations of congenital diseases, traumatic injuries or tumour ablation, severe loss of skeletal muscle may occur with consequent impairments in aesthetics and functions. Volumetric muscle loss (VML) is defined as the defect size exceeds the endogenous regeneration capacity.^3^, ^4^

The standard of clinical management for VML is the autologous transfer of muscle flaps or grafts. This approach, however, is limited by the tissue availability and morbidity in the donor site. As a result, novel strategies based on tissue engineering and regenerative medicine principles are of tremendous potential in improving the prognostic of VML.^5^, ^6^ Complete function recovery is the ultimate goal of VML repair, and a reliable VML model with distinct function impairment is indispensable in the exploration of effective intervention strategies.

The orofacial region harbours over 60 pieces of muscles that orchestrate intricate fine movements and diverse physiological functions, including feeding, breathing, eye movement and facial expression.^7^, ^8^ These critical functions may be impaired when VML occurs. Compared with muscles in the limbs and trunk, orofacial muscles are of distinct embryonic origins and response to injury.^7^, ^9^, ^10^, ^11^ Studies in vertebrate models showed that the molecular regulation during development is different in orofacial muscles from limb and trunk muscles.^11^ The mesenchymal cells in orofacial muscles were of neural crest origin while those in the limb and truck were of mesoderm.^11^, ^12^

In addition, as compared with the limb and truck counterparts, orofacial muscles host fewer satellite cells and demonstrate more severe and persistent fibrosis after injury.^12^, ^13^, ^14^ Bearing such heterogeneity in mind, a VML intervention that works in the limb/trunk still needs to be tested for effectiveness in the orofacial muscles. Currently, however, no reliable orofacial VML model with concrete functional correlation is available for such a purpose.^4^, ^15^, ^16^, ^17^, ^18^

In this study, we introduced defects to the major masticatory muscle masseter and successfully induced persistent fibrosis and consequent deficit in mastication on nutrition, providing a reliable and convenient mouse model for testing strategies of orofacial VML recovery.

## 2 MATERIALS AND METHODS

### 2.1 Mice

This study was conducted in compliance with the Animal Welfare Act and the Implementing Animal Welfare Regulations and in accordance with the principles of the Guide for the Care and Use of Laboratory Animals. All animal procedures were approved by the ethics board of West China Hospital of Stomatology, Sichuan University (Protocol No. WCHSIRB‐D‐2020‐114). Wild‐type 6‐week‐old male C57BL6/J mice were housed in pathogen‐free conditions with food and water ad libitum.

### 2.2 Masseter VML injury and autologous graft transplantation

**FIGURE 1:**
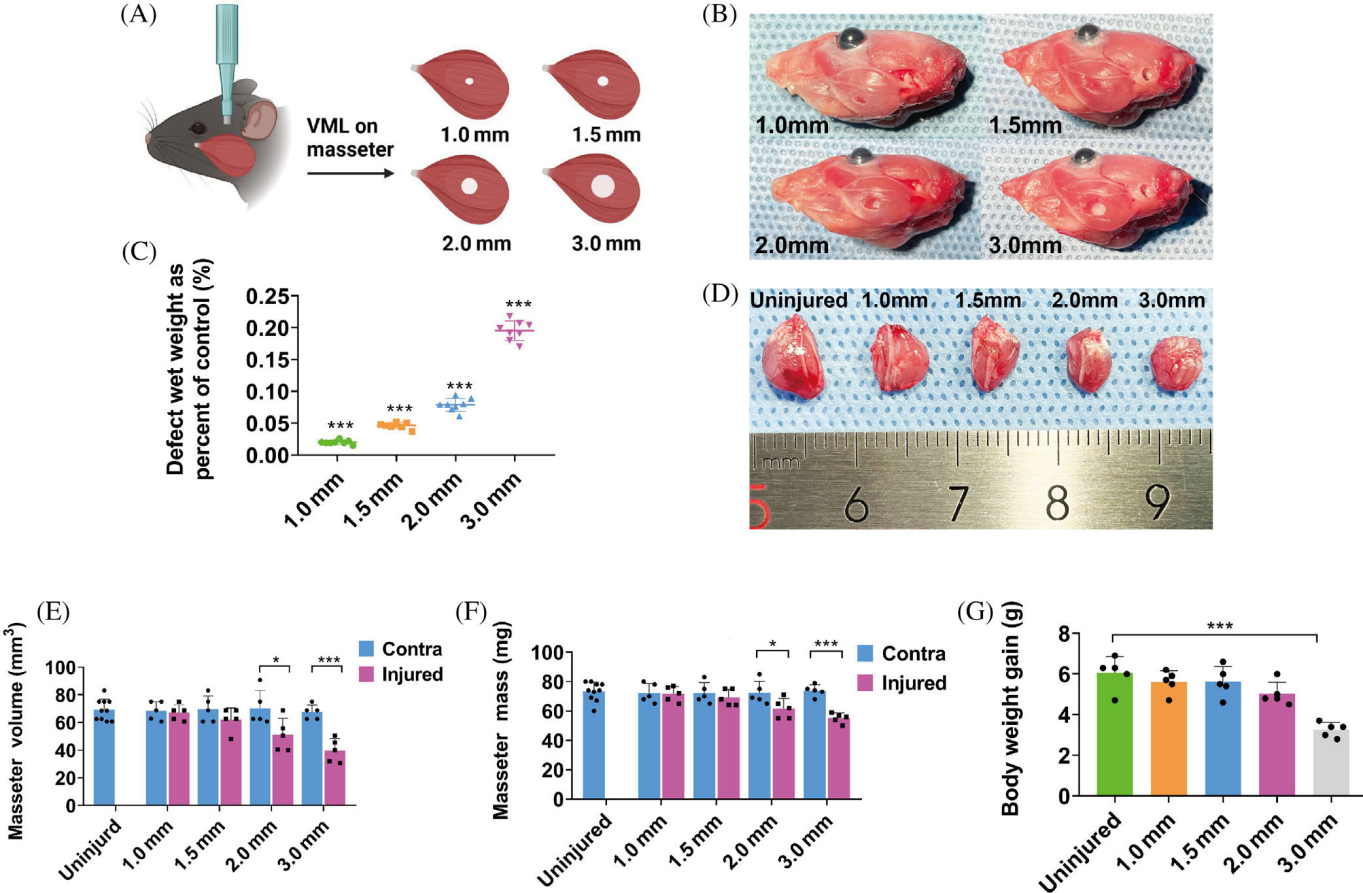
VML injuries in mouse masseter muscle. (A, B) Schematic diagram and photos showing masseter VML injuries of different diameters. (C) The wet weight of biopsied masseter muscles as a percentage of the contralateral intact masseter for each biopsy punch size. Each biopsy size percentage is compared with every other biopsy punch size percentage (*n* = 8). (D) The gross morphology of the injured masseters at PID 28. (E, F) Wet weight and volume of the injured masseter measured at PID 28 (*n* = 5). (G) Body weight gain of experimental animals during the 28 days (*n* = 5). One‐way ANOVA was used for statistical analysis. p* < 0.05, ***p < 0.001. Contra, contralateral control; ns, not significant; PID, post‐injury day; VML, volumetric muscle loss.

Mice were anaesthetised through inhalation of 1%–3% isoflurane. Sustained release buprenorphine (0.8 mg/mL) was given for pain management. The maxillofacial incision was depilated and sterilized with ethanol and iodophor. All operations are performed by a single surgeon. The body weight of the mice was weighed prior to surgery. An incision was made to expose the masseter muscle. A full‐thickness defect was created in the mid‐belly region of the masseter muscle up to the bone surface using 1.0, 1.5, 2.0 or 3.0 mm diameter biopsy punching tools (Miltex, 15110‐10, −15, −20, −30) (Figure 1A, B). Intraoperative injury of facial blood vessels and nerves should be avoided. The deeper fascia and surface skin layers were separately closed using an interrupted stitch with a 5‐0 absorbable suture (Vicryl, Ethicon, Summerville, USA). The muscle tissue removed from the defect site was weighed. The contralateral masseter was left uninjured to serve as a comparative control. Mice used for masticatory function evaluation had VML injuries on both masseter muscles. A sterile surgical pen was used to draw an orientation line marking the natural alignment of masseter muscle myofibers in the donor and recipient sites, respectively. After that, the masseter muscle autograft was performed according to the direction line. For autogenous muscle grafts, muscle plugs were immediately implanted in the contralateral defect site. The transplanted muscle myofibers were aligned with the surrounding masseter muscle myofibers. All animals were housed for a 28‐day recovery period.

### 2.3 Mastication rate measurement

Analysis of mastication behaviours was based on methods described in previous studies.^19^ Briefly, food was removed from mice for 14–16 h overnight. After that, one food pellet was reintroduced. The eating behaviour of each mouse was recorded from the lateral view by a video camera (CANON EOS 60D, Tokyo, Japan). Videos were recorded as MP4 files at 60 frames/s and analysed by two blinded independent reviewers using AVIDEMUX 2.6.0 software. Each chewing episode started with maximum jaw closure, and the number of consecutive chewing cycles (maximum jaw opening and then complete closure again) was counted for the next 59 consecutive viewing frames to obtain the chewing rate (number of chewing cycles per second). Mastication rates were determined by counting five independent, consecutive chewing clips from each animal in 60 frames of video. Data are for the average number of chews per second per mouse. After recording, each mouse was returned to its cage and given free food and water.

### 2.4 Bite force measurement

**FIGURE 2:**
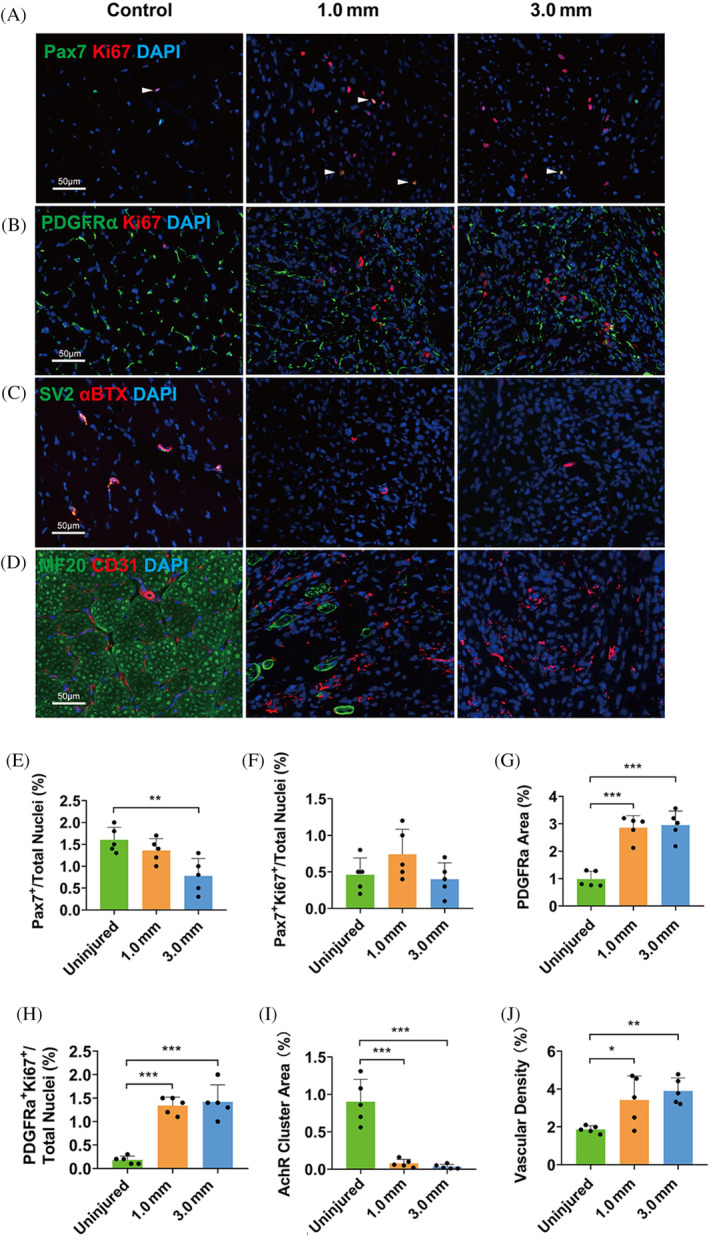
Cells infiltration and proliferation in mouse masseter VML model at PID14. (A, E, F) Infiltration and proliferation of MuSCs in 1.0 and 3.0 mm diameters (*n* = 5). (B, G, H) Infiltration and proliferation of FAPs in 1.0 and 3.0 mm diameters (*n* = 5). (C, I) AChRs clusters for αBTX positive (red) and synaptic vesicle for SV2 positive (green), AChRs clusters of the double positives in the damaged area were counted for quantitative analysis of NMJ (*n* = 5). (D, J) Infiltration of endothelial cells in 1.0 and 3.0 mm diameters (*n* = 5). One‐way ANOVA was used for statistical analysis. p* < 0.05, **p < 0.01, ***p < 0.001. FAPs, fibroadipogenic progenitors; PID, post‐injury day; MuSCs, muscle stem cells.

We used the FlexiForce sensor (B201‐L‐8) and the Economic Load and Force (ELF) system from Tekscan (South Boston, USA) to measure bite force. Prior to testing, mice were housed in a custom‐made clear plastic cage with a small gap for 30 min to allow them to acclimate (Figure 2B). The sensor is coated with hard plastic and calibrated linearly by loading a series of graduated weights (ranging from 1 ~ 1000 g). According to the standard protocol for maximal bite force testing,^20^ we measured the animals for 1 min without painful stimuli and recorded the raw data continuously for analysis. The same tests were carried out five times per mouse, with 3 min between each test. The maximum bite force (in grams) for each animal was recorded for each trial and the mean of all trials was then calculated.

### 2.5 Eating behaviour analyses

Spontaneous eating behaviour was evaluated by EthoVision software (Noldus, Wageningen, Netherlands). The software could automatically detect and recognize a variety of mouse behaviours, including eating, drinking, grooming, jumping, supporting upright, non‐supporting upright, curling, sniffing, walking and resting.^21^, ^22^, ^23^, ^24^, ^25^, ^26^ In this study, mice were habituated to the device environment three times prior to measurement. On the day of the experimental procedure, mice were kept on the experimental platform for 15 h from 6:00 PM to 09:00 AM the following day. The EthoVision XT can detect animals in video files or live video, distinguish them from the background, and track their whereabouts and movements. As the animal consumed food, the software recorded these behaviours and analysed the timing and frequency of eating. The amount of food intake was also recorded.

### 2.6 Masseter harvest and preparation for histology analysis

Mice were sacrificed 28 days after VML with an overdose of isoflurane. The body weight of the mice was weighed before harvesting. The masseter muscle consists of a superficial and a deep part, and some of the superficial bundles are also inserted into the aponeurosis of the deeper masseter layer, making it often difficult to separate these two parts of the masseter muscle.^27^ Therefore, we made the method of masseter muscle acquisition consistent. After removing the skin from the angle of the mouth to the parotid gland, the superficial masseter tendon was exposed at the small nodule at the lower margin of the infraorbital foramen. The tendon was cut, and the muscle was sharply separated along the mandibular surface until an intact masseter muscle was obtained. Facial nerve injury should be avoided during the operation. The mass of the masseter muscle was weighed, with values accurate to one decimal place. And, the muscle tissue volume was calculated by using the formula: volume (*V*) = (tissue length) × (tissue width)^2^/2.^28^

The masseter was then inserted into a cylindrical mould made of tinfoil, embedded in a freezing solution (made of two parts Tissue Tek OCT compound and 1 part 30% sucrose deionized water) and frozen in liquid nitrogen‐cooled isopentane (Macklin, Shanghai, China).^12^ Frozen sections 10 mm thick from the mid‐portion of the defect site were cut using a Leica cryostat and then fixed in acetone (KESHI, Chengdu, China) at 0°C for 15 min and then dried in a fume hood at room temperature for 20 min.

### 2.7 Histological and morphometric analysis

To observe muscle histological alterations, muscle sections were stained with haematoxylin and eosin (H&E) following the manufacturer's instruction. Sirius red staining following the manufacturer's instruction was used to observe the area of collagen deposition, and polarized light microscopy was used to analyse the area of type I collagen (red) and type III collagen (green). Laminin & DAPI staining was used to assess the regeneration of muscle fibres in the central region of the injury. Image J was used to analyse the collagen deposition area, fibre number, fibre diameter and fibre cross‐sectional area of the masseter muscle slices under the 20× field of view in the area of maximum damage.

### 2.8 Immunofluorescence

After washing and rehydration in phosphate‐buffered saline (PBS), sections were blocked and permeabilized with blocking buffer (5% bovine serum albumin, 5% donkey serum, 0.3% Triton‐X in 1× PBS) for 1 h, then labelled with primary antibodies overnight at 4°C and incubated with secondary antibody for 1 h at 25°C. Nuclei were stained with DAPI. Immunofluorescence was imaged with an Olympus VS200 microscope (Olympus Corporation, Tokyo, Japan).

The primary and secondary antibodies applied were as below: rabbit anti‐laminin polyclonal antibody (L9393; dilution:1:600; Sigma‐Aldrich), mouse anti‐Pax7 monoclonal antibody (AB_528428; dilution:1:5; DSHB), rabbit anti‐Ki67 polyclonal antibody (ab15580; dilution:1:400; Abcam), goat anti‐PDGFRα polyclonal antibody (AF1062; dilution:1:40; R&D Systems), mouse anti‐SV2 monoclonal antibody (AB_2315387; dilution:1:10; DSHB), cy3 α‐bungarotoxin (B00018; dilution:1:500; Bosunlife), rabbit anti‐CD31 polyclonal antibody (ab281583; dilution:1:50; Abcam), mouse anti‐MF20 monoclonal antibody (MAB4470; dilution:1:2; DSHB), donkey anti‐rabbit secondary antibodies, (A2442; dilution:1:200; IFKine), donkey anti‐goat secondary antibodies, (ab150129; dilution:1:200; Abcam).

### 2.9 Statistical analysis

Statistical analyses were completed using GraphPad Prism version 8.0.1 software. One‐way analysis of variance (ANOVA) for comparisons among multiple groups with one variable was used. *p* < 0.05 was considered significant. All data are presented as the mean ± standard deviation.

## 3 RESULTS

### 3.1 Development of a critical size mouse masseter VML injury

To determine the critical size of masseter VML injury with masticatory function impairment, biopsy punches of 1.0, 1.5, 2.0 and 3.0 mm diameters were used to make full‐thickness defects in the centre of mouse masseter (Figure 1A,B), removing approximately 2%, 5%, 8% and 20% of the total masseter wet weight respectively (Figure 1C). At post‐injury day (PID) 28, a significant decrease in masseter volume and mass was observed among animals receiving VML of 2.0 and 3.0 mm diameters (Figure 1D–F). There was no significant difference in mass or volume between uninjured contralateral and age‐matched control muscles, indicating that VML did not have a significant impact on the contralateral masseter muscle (Figure 1E,F). Following a 28‐day period, only VML of 3.0 mm diameter resulted in a significant decrease in body weight gain as compared with the age‐matched intact control (Figure 1G), suggesting 3.0 mm had an effect on the overall health of the mice.

### 3.2 Proliferative activity of muscle repair effector cells

The proliferative activity of muscle stem cells (MuSCs) and mesenchymal fibroadipogenic progenitors (FAPs) were monitored at PID14 among animals receiving 1.0 and 3.0 mm diameter defects (Figure 2A,B). The percentage of Pax7^+^ MuSCs after a 1.0 or 3.0 mm defect was significantly lower than the control, but no significant difference in the proliferation of MuSCs between the intact control and the defect groups (Figure 2E,F). Both the PDGFRα^+^ FAPs area and the proliferative level of FAPs increased significantly at PID14 as compared with the control (Figure 2G,H). The density of neuromuscular junction (NMJ) was significantly lower after defect, as indicated by immunostaining of acetylcholine receptors (AChRs) clusters (Figure 2C,I), while the vascular density as indicated by CD31 labelling was significantly increased after defect injury (Figure 2D,J).

### 3.3 Masseter muscle histological alterations

**FIGURE 3:**
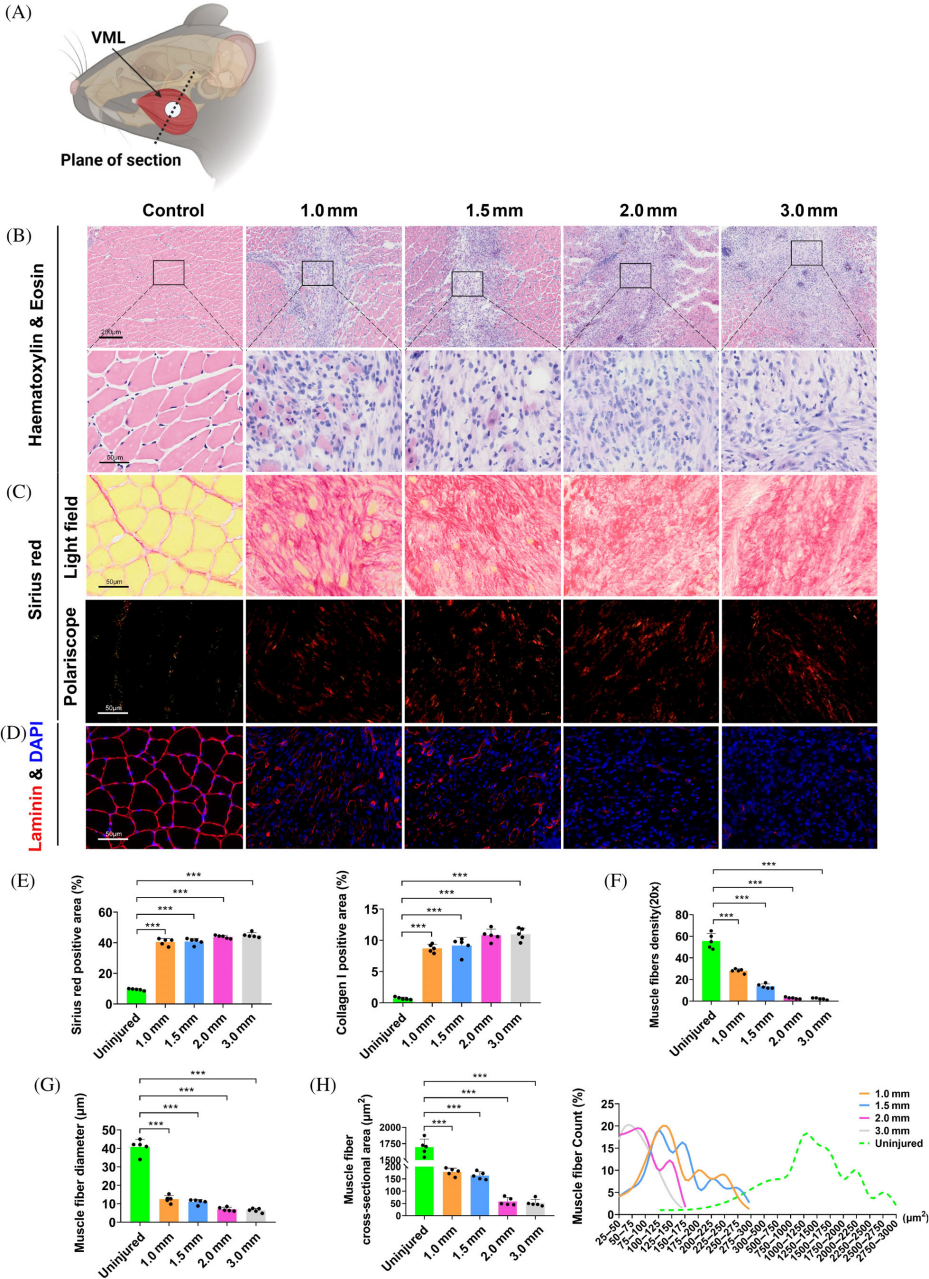
Histologic analyses of masseter muscles 28 days after VML injuries. (A) Schematic diagram indicating the orientation of tissue sectioning. (B–D) Representative Haematoxylin & Eosin staining, Sirius red staining and Laminin immunostaining at PID28 (*n* = 5). Quantitative comparison in fibrosis and myogenesis was performed using measurements including the severity of fibrosis (E, *n* = 5), muscle fibre density (F, *n* = 5), muscle fibre diameter (G, *n* = 5), muscle fibre cross‐sectional area, and muscle fibre area frequency distribution (H, *n* = 5). One‐way ANOVA was used for statistical analysis. ***p < 0.001. PID, post‐injury day; VML, volumetric muscle loss.

Both fibrosis and myogenesis status were examined in the centre region of VML injuries at PID28 (Figure 3A). Persistent fibrosis was observed in all injured groups, and only scattered myofibers were noted among samples of 1.0 and 1.5 mm diameter VML (Figure 3B). In contrast, mouse tibialis anterior muscles receiving the same VML injuries demonstrated significantly less fibrosis and more robust myogenesis, characterized by regular myofiber alignment with central nuclei, which is consistent with previous observations (Figure S1).^11^, ^12^, ^13^ Masseter VML samples all demonstrated significant increase in the percentage of sirius red positive area and collagen I deposition area as compared with the intact controls, but no significant difference was noted among VML groups (Figure 3C,E). The *de nove* myofibers in the VML region were visualized using Laminin immunostaining. VML injuries of all sizes resulted in a significant decrease in the density, diameter and cross‐sectional area of myofibers. Besides, these measurements were significantly lower among animals receiving 2.0 and 3.0 mm diameter VML than those receiving 1.0 and 1.5 mm (Figure 3D,F–H). In addition, we explored the effect of unilateral masseter VML on the wear of teeth and temporomandibular joints but found no significant pathological changes or differences between each side (Figures S2 and S3). These data provide a comprehensive reference for selecting the most appropriate VML sizes for testing specific interventions.

### 3.4 In situ masseter muscle functional characteristics

**FIGURE 4:**
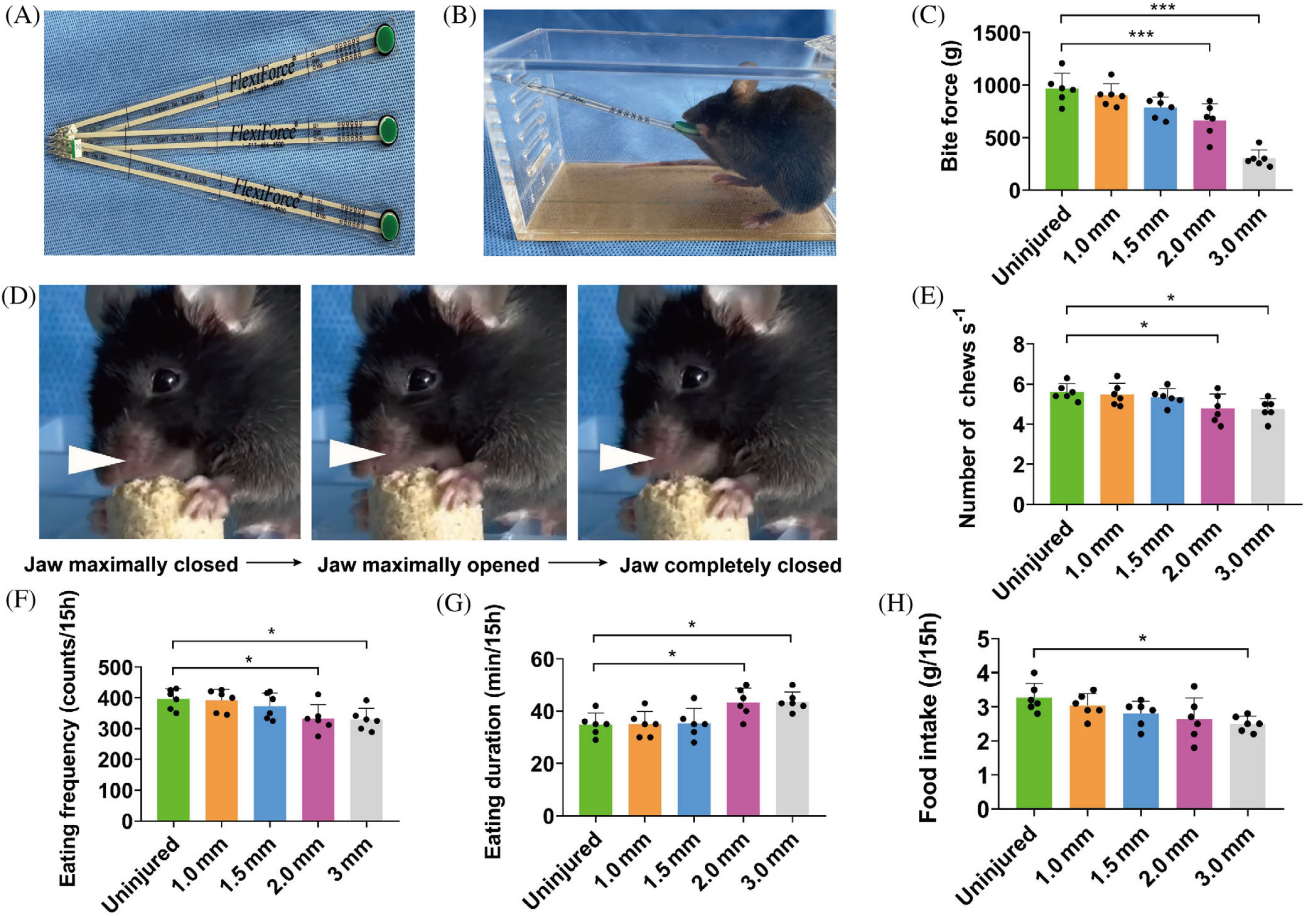
Masticatory function analyses after mouse masseter VML injury. (A, B) The FlexiForce sensors from Tekscan were used for bite force measurement. (C) The bite force measured at PID28 among animals with VML of different sizes (*n* = 6). (D) Single masticatory episode depicted by still frames from a representative masticatory assay video. (E) Mastication rate among animals with VML of different sizes (*n* = 6). (F, G) The food intake was recorded for 15 h and the eating duration and frequency were recorded using EthoVision XT tracking software (*n* = 6). (H) The amount of food intake during the 15‐h recording (*n* = 6). One‐way ANOVA was used for statistical analysis. p* < 0.05, ***p < 0.001. PID, post‐injury day; VML, volumetric muscle loss.

At PID28 a series of behavioural tests on mastication were performed, including bite force test, masticatory rate test and spontaneous feeding behavioural assessment. The experiment setup for bite force measurement is shown in Figure 4A,B. A single mastication episode was illustrated in Figure 4D. As compared with the uninjured controls, a significant decrease in bite force and masticatory rate was observed among animals receiving masseter VML in diameter of 2.0 or 3.0 mm (Figure 4C,E). Among animals receiving VML of 3.0 mm diameter, the bite force was less than one‐third of that of uninjured controls (Figure 4C). The feeding activities of the experimental animals were recorded continuously for 15 h. In consistent with the bite force and mastication rate data, a significant decrease in eating frequency and an increase in eating duration were observed among mice receiving VML of 2.0 or 3.0 mm diameters (Figure 4F,G). In addition, the overall food intake during the 15 h of recording was significantly less among mice with VML of 3.0 mm diameter (Figure 4H), which was consistent with the less body weight gain in this group.

### 3.5 Autologous muscle grafts for the VML model

**FIGURE 5:**
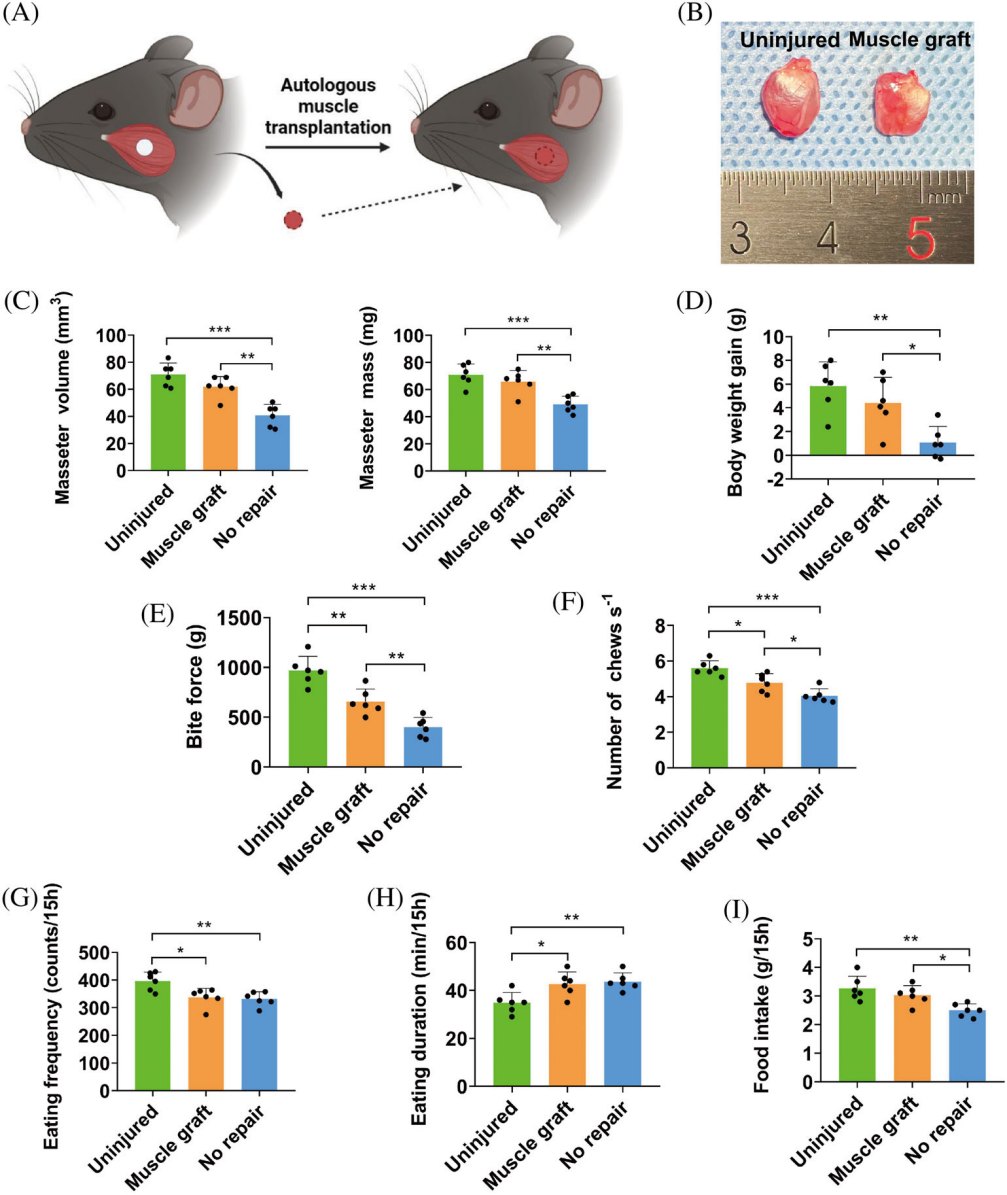
Testing the effectiveness of autologous muscle transplantation in mouse masseter VML model. (A) Diagram showing immediate transplantation of autologous muscle graft repairing masseter VML injury. (B) The gross morphology of the muscles 28 days after autologous muscle graft. (C) Wet weight and volume of the masseters measured at PID28 (*n* = 6). (D) Body weight gain was measured to evaluate the effect of the muscle graft repair on nutrition intake (*n* = 6). (E–I) Functional analysis of muscle transplantation repair group (*n* = 6). One‐way ANOVA was used for statistical analysis. p* < 0.05, **p < 0.01, ***p < 0.001.

Lastly, we tested the effectiveness of autologous muscle grafts on the 3.0 mm masseter VML model (Figure 5A). Muscle grafts harvested by biopsy punch were immediately transferred to the masseter on the contralateral side. At PID28, autologous grafts effectively relieved reduction in masseter volume, masseter mass (Figure 5B,C) and body weight gain (Figure 5D) after VML injury. Accordingly, animals receiving autologous grafts demonstrated superior bite force, higher mastication rate and more food intake as compared with those without grafts, though not achieving the level of uninjured controls (Figure 5E–I).

**FIGURE 6:**
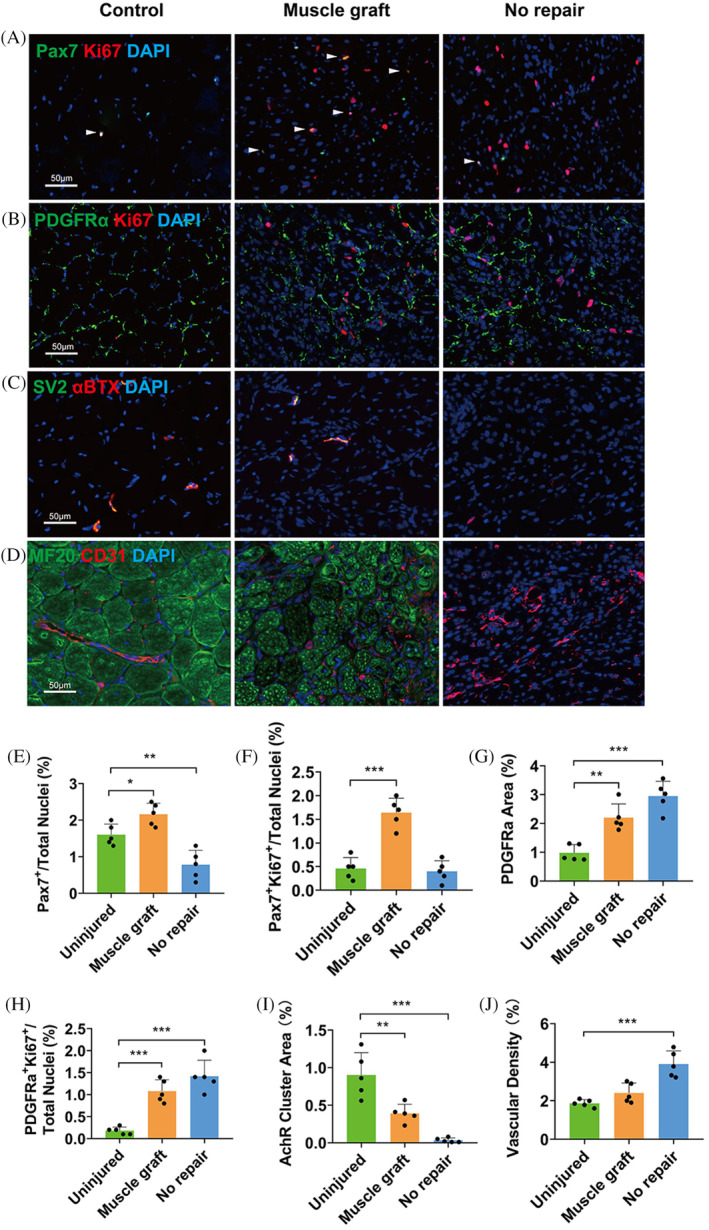
Cells infiltration and proliferation in the region of autogenous muscle grafts at PID14. (A, E, F) Infiltration and proliferation of MuSCs in autogenous grafts (*n* = 5). (B, G, H) Infiltration and proliferation of FAPs in autogenous grafts (*n* = 5). (C, I) Clustering of AChRs in autogenous grafts (*n* = 5). (D, J) Infiltration of endothelial cells in autogenous grafts (*n* = 5). One‐way ANOVA was used for statistical analysis. p* < 0.05, **p < 0.01, ***p < 0.001. FAPs, fibroadipogenic progenitors; MuSCs, muscle stem cells; PID, post‐injury day.

Among the animals receiving autologous graft, the percentage of MuSCs in the defect region was comparable to intact control and the proliferation of MuSCs was significantly activated (Figure 6A,E,F). No significant difference was observed in the FAPs percentage or proliferation between animals with or without autologous grafts (Figure 6B,G,H). Autologous grafts maintained part of their NMJs, but the density was still significantly lower than in the intact tissue (Figure 6C,I). Besides, the vascular density was lower in the grafted animals than those without (Figure 6D,J).

**FIGURE 7:**
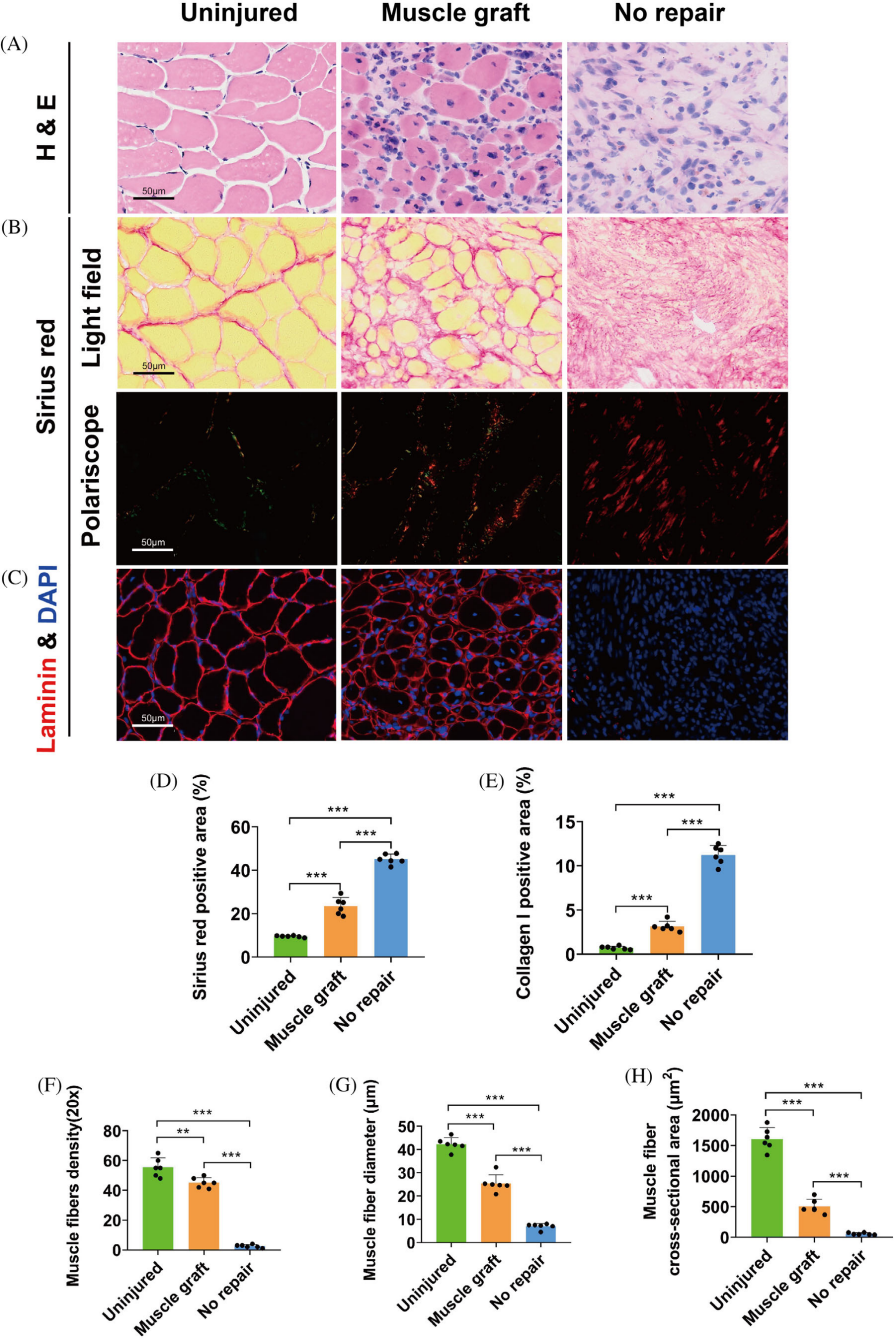
Histological analysis of autogenous muscle grafts in mouse masseter VML model. (A, B) Representative Haematoxylin & Eosin staining, Sirius red staining of muscle graft repair 28 days post VML. Sirius red was stained for the analysis of Sirius red positive area and collagen I positive area (D, E, *n* = 6). (C) Representative Laminin & DAPI staining of muscle graft repair 28 days post VML. Sections were analysed for muscle fibre density, muscle fibre diameter, and muscle fibre cross‐sectional area under a 20× field of view (F–H, *n* = 6). One‐way ANOVA was used for statistical analysis. **p < 0.01, ***p < 0.001.

Further histological analysis showed that animals with autologous grafts relieved the severity of fibrosis and collagen I deposition in the VML region by up to 50%–70% as compared with those without grafts (Figure 7A,B,D,E). Accordingly, the group with graft demonstrated muscle fibres of significantly higher density, diameter and area than the group without (Figure 7C,F–H). These data supported the effectiveness of autologous muscle graft in repairing orofacial VML while at the same time indicated space for further improvement.

## 4 DISCUSSION

Reliable and clinically relevant experimental models are critical to the understanding of pathological mechanisms and the development of novel interventions. Previously reported VML models were established on limb/trunk,^15^, ^16^, ^17^, ^18^, ^29^ where numerous tissue engineering and regenerative medicine strategies for muscle repair were tested. The trunk and limb muscles develop from the thoracic and lumbal somites, while the orofacial muscles develop from the mesoderm of the pharyngeal arches and the occipital somites.^11^ The molecular regulation of orofacial muscle development also differs from that of trunk and limb muscles.^11^ In addition, the regenerative ability of orofacial muscles is lower, and they develop more fibrosis than trunk limbs and muscles.^11^ The regeneration of muscles is orchestrated by a blended source of stem and progenitor cells, mainly including MuSCs and FAPs.^30^ Limb muscles host MuSCs of the Pax3 lineage, and FAPs from the mesoderm, while orofacial muscles have MuSCs of the Mesp1 lineage and FAPs from the ectoderm‐derived neural crest.^30^ Considering the particularity of orofacial muscle in embryonic origin, injury response and physiological function^7^, ^9^, ^10^, ^11^, ^13^, ^31^, ^32^ interventions effective in the limb/trunk still need to be tested in the orofacial region. This study proposed the first injury model on orofacial muscle with satisfactory reliability, convenience and clinical relevance. A full‐thickness tissue punch of 3.0 mm diameter in the mouse masseter was sufficient to cause concrete persistent fibrosis phenotype, decrease in muscle mass, deficit in masticatory performance and eventually limited nutrition intake, successfully mimicking the injury‐induced mastication dysfunction observed in the clinics.

Our model selected the masseter for its superficial location and critical role in mastication. The masseter is the dominant jaw‐closing muscle in the Rodentia, comprising between 60% and 80% of the entire masticatory muscle mass27 and contributing the most bite force.^33^, ^34^ In humans, the masseter is also the primary chewing muscle that produces the most powerful force, and the size of the masseter muscle is positively correlated with bite force.^33^ The size of the masseter is regarded as a determining factor in chewing force and function.^34^, ^35^ Our VML model induced a significant reduction in masseter mass and volume, as long as loss in function.

This study further provided a protocol for histological and functional analyses of the model. The status of fibrosis and myogenesis was evaluated with previously reported tissue staining techniques. The function of the masseter was measured by mastication rate, bite force and spontaneous feeding behaviour.^19^, ^36^ Mastication rate was based on rhythmic cycles of jaw opening and closing. Two stages of mastication movement have previously been described for mice, as incision and chewing which correspond to transport (deliver the ingested material from the incisors to the molars) and processing (reducing the food to a swallowable consistency through molar chewing and salivary mixing) described in human.^37^, ^38^ The incision movements are rapid, small and irregular, making it difficult to analyse. In comparison, chewing movements are slower, more obvious and rhythmic. Thus, chewing cycles instead of incision cycles were analysed in this model. The mean mastication rates of uninjured mice were 5.6 Hz in this study, which was consistent with previously published findings in wild‐type uninjured mice (5.5 Hz).^19^

Recently, several innovative methods were developed to measure bite force.^39^, ^40^, ^41^ In this study, we used the FlexiForce sensors which have been shown to be feasible in bite force measurement among 13 small mammal species.^20^ In addition, we used a specially designed plastic cage with multiple small slots on the side wall (Figure 4B), which allowed the insertion of the sensor for the mice to bite. The modest space of the cage allowed the mice to be unrestrained but still during testing, avoiding resistance to bite after forced restraint. We presented the sensor to the mice and recorded the force of a single bite. When several consecutive rapid bites were recorded, the maximum value was used for analyses.^20^ Our results indicated that the reduction in the bite force was accordant with the extent of reduction in masseter muscle mass and volume.

Eating patterns, including eating frequency and duration, were recorded using the EthoVision XT system, which is the most widely applied tracking software that tracks and analyzes the activity of animals.^21^, ^22^, ^23^, ^24^, ^25^, ^26^ It distinguishes animals from the background in the video and tracks their whereabouts and movements. The automatic behaviour recognition is precise, reliable and tireless, overcoming the shortcomings of traditional manual observation.^26^, ^42^ We used the eating module in the automatic behaviour recognition system to detect eating duration and frequency. Our results indicated that VML injuries of 2 mm or greater showed a significant increase in eating duration and decrease in eating frequency, suggesting that the injured masseter muscles were prone to fatigue and resulted in inefficient mastication. In addition, 3 mm VML injury showed a significant decrease in the amount of food intake and consequently a decrease in body weight gain.

In an analysis of critical effector cells to muscle repair and regeneration, we found that both 1.0 and 3.0 mm injuries resulted in reduced infiltration of MuSCs and reduced AChRs clusters but increased FAPs in the defect area, which is consistent with our finding of persistent fibrosis and masticatory function impairment at PID28. The 1 mm defect demonstrated upregulation in both myogenesis and fibrosis, but the 3 mm defect demonstrated no obvious myogenic cell activity. The possible reason is that the repair effector cells in the surrounding muscle tissue of the 1 mm injuries were more likely to migrate to the area of injuries for repair, while the repair cells of the larger injuries could not infiltrate the area of injuries. Autologous grafts significantly increased MuSCs presence and proliferation, as well as the density of NMJs. Therefore, autologous transplantation, to a certain extent, alleviates the degree of fibrosis and improves the masticatory function at PID28.

Unilateral mastication pattern is a manifestation of impaired mastication function,^42^ which can be caused by pain and impairments.^43^, ^44^, ^45^ There is increasing evidence that prolonged unilateral mastication adversely affects maxillofacial soft and hard tissues due to the presence of asymmetric forces.^46^, ^47^, ^48^, ^49^ In this study, however, we did not observe any significant damage to the articular cartilage, trabecular bone of the condyle or tooth following unilateral masseter VML in a period of 28 days. The mastication behaviour changes may be fully compensated by the dentoskeletal system. In future studies, we will focus on greater unilateral masseter VML injuries and longer postoperative time point to determine the impact on TMJ and teeth.

Although transplantation of autologous muscle flap is the current gold standard for the clinical management of VML, it is often associated with donor‐site morbidity, limited tissue availability, and complications such as infection and necrosis.^5^ In this study, the masseter VML was grafted with muscle from the opposite side masseter immediately after injury. The muscle graft effectively reduced the severity of fibrosis and functional impairment, as compared with the animals without graft, but still failed to achieve complete regeneration and functional recovery, indicating space for further improvement, especially with the aid of advances in tissue engineering and stem cell medicine.^50^, ^51^, ^52^

Collectively, we presented for the first time an injury model in the orofacial masticatory muscle with function impairment and systemic impact, along with a comprehensive panel of quantitative histological and functional analyses, providing a powerful tool for testing and developing tissue engineering and regenerative medicine approaches for orofacial muscle repair.

## AUTHOR CONTRIBUTIONS

JL and BS conceived the study. NZ and YH performed most of the experiments. NZ, YH, XC, LX and WX contributed to the analysis of the data. NZ and JL discussed the results and wrote the article. All authors read and approved the final article.

## FUNDING INFORMATION

This work was supported by the National Natural Science Foundation of China (Grant No. 82001031and 81974147) and the Research and Develop Program, West China Hospital of Stomatology Sichuan University (RD‐03‐202007).

## CONFLICT OF INTEREST STATEMENT

The authors declare no conflict of interest.

## Supporting information


**Figure S1.** Representative images of haematoxylin and eosin (H&E) and sirius red staining in tibialis anterior muscle cross‐sections at 28‐day recovery after VML. (A) Compared with masseter muscle, tibialis anterior muscle demonstrated stronger regeneration ability after VML. (B–E) Sections were analysed for sirius red positive area, muscle fibre density, muscle fibre diameter, and muscle fibre cross‐sectional area under a 20× field of view. One‐way ANOVA was used for statistical analysis. ***p < 0.001.

**Figure S2.** Compare the bilateral condylar cartilage and condyles trabecular bone 28‐day recovery after unilateral VML. (A) No significant differences between L and R condyle cartilage in Alcian blue and haematoxylin & eosin staining (*n* = 5). (C) cartilage area, (D) OARSI score, (E) cartilage thickness. (B) No significant differences between L and R condyles of trabecular bone. Student's *t*‐test was used for statistical analysis. L, left; ns, not significant; R, right.

**Figure S3.** Compare the bilateral tooth wear 28‐day recovery after unilateral VML. No significant differences between L and R tooth wear. L, left; R: right.

**Data S1.** Supporting information.

**Data S2.** Supporting information.

## Data Availability

The data that support the findings of this study are available from the corresponding author upon reasonable request.
