# Supplementary figures and images for: A critical size volumetric muscle loss model in mouse masseter with impaired mastication on nutrition
Source: Cell Prolif. 2024 Feb 14;57(6):e13610. doi: 10.1111/cpr.13610 (PMC11150142; doi:10.1111/cpr.13610)

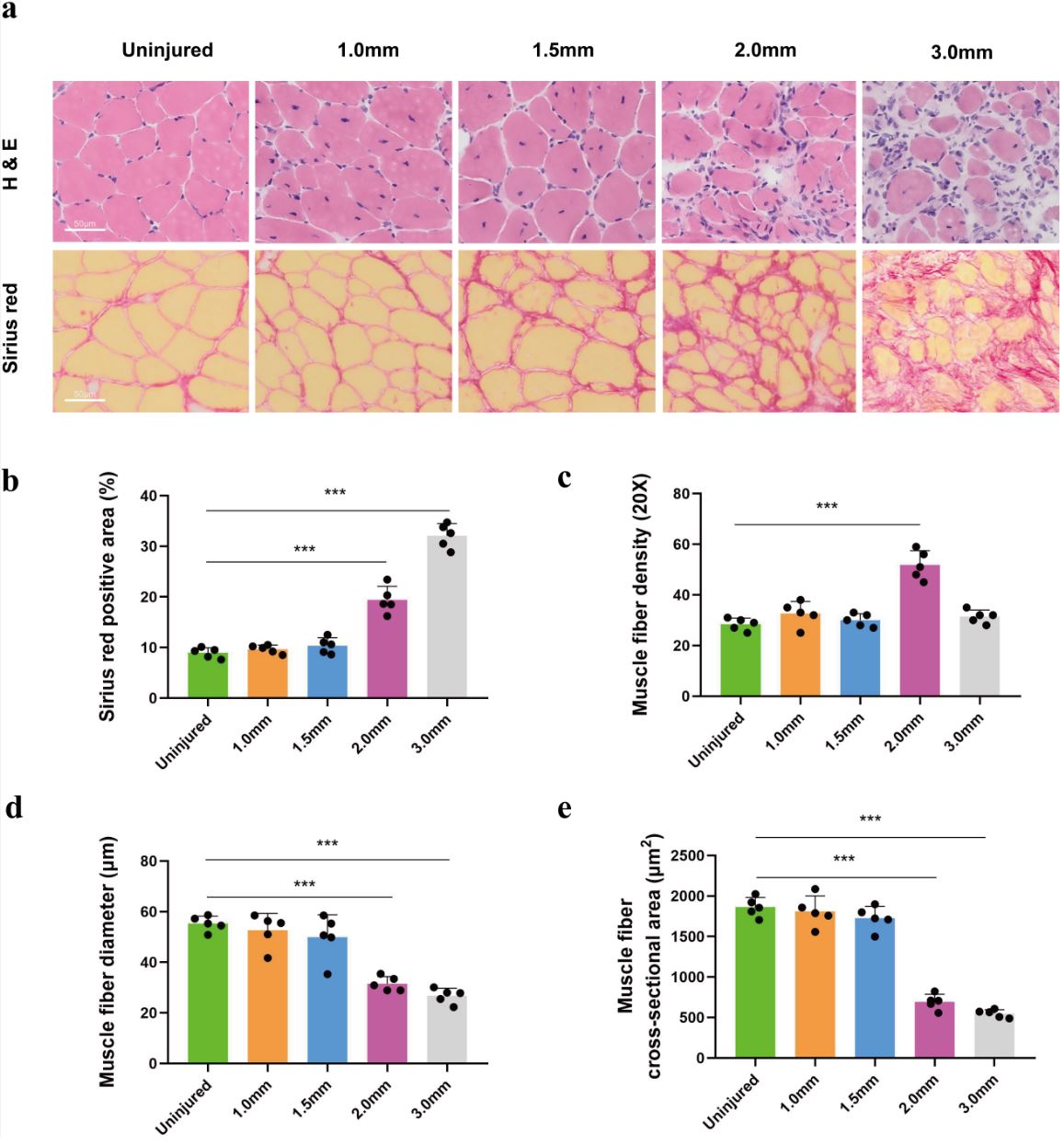

Supplement: Supplementary file 1 — Figure S1. Representative images of haematoxylin and eosin (H&E) and sirius red staining in tibialis anterior muscle cross‐sections at 28‐day recovery after VML. (A) Compared with masseter muscle, tibialis anterior muscle demonstrated stronger regeneration ability after VML. (B–E) Sections were analysed for sirius red positive area, muscle fibre density, muscle fibre diameter, and muscle fibre cross‐sectional area under a 20× field of view. One‐way ANOVA was used for statistical analysis. ***p < 0.001. [file CPR-57-e13610-s001.JPG]

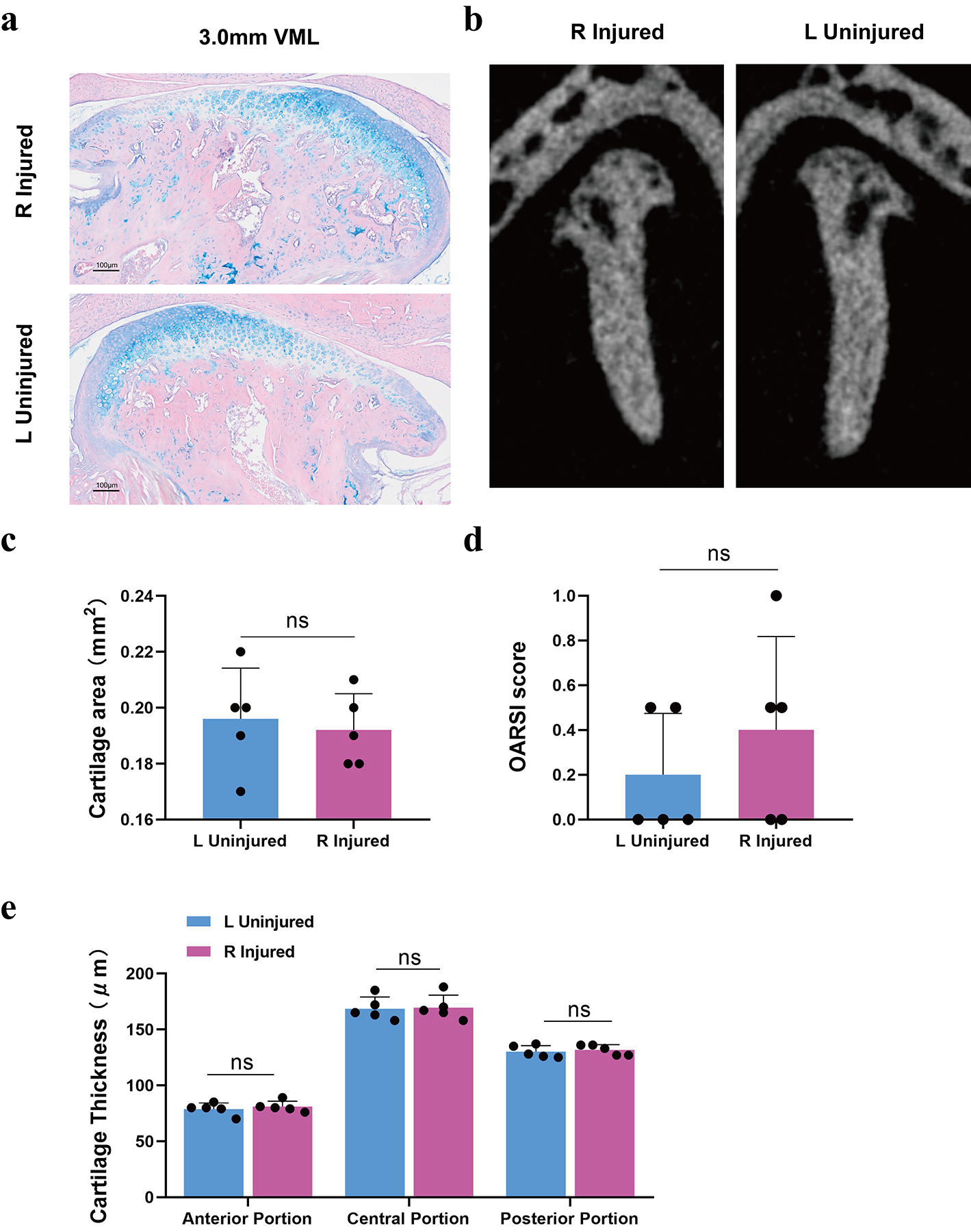

Supplement: Supplementary file 2 — Figure S2. Compare the bilateral condylar cartilage and condyles trabecular bone 28‐day recovery after unilateral VML. (A) No significant differences between L and R condyle cartilage in Alcian blue and haematoxylin & eosin staining (n = 5). (C) cartilage area, (D) OARSI score, (E) cartilage thickness. (B) No significant differences between L and R condyles of trabecular bone. Student's t‐test was used for statistical analysis. L, left; ns, not significant; R, right. [file CPR-57-e13610-s005.jpg]

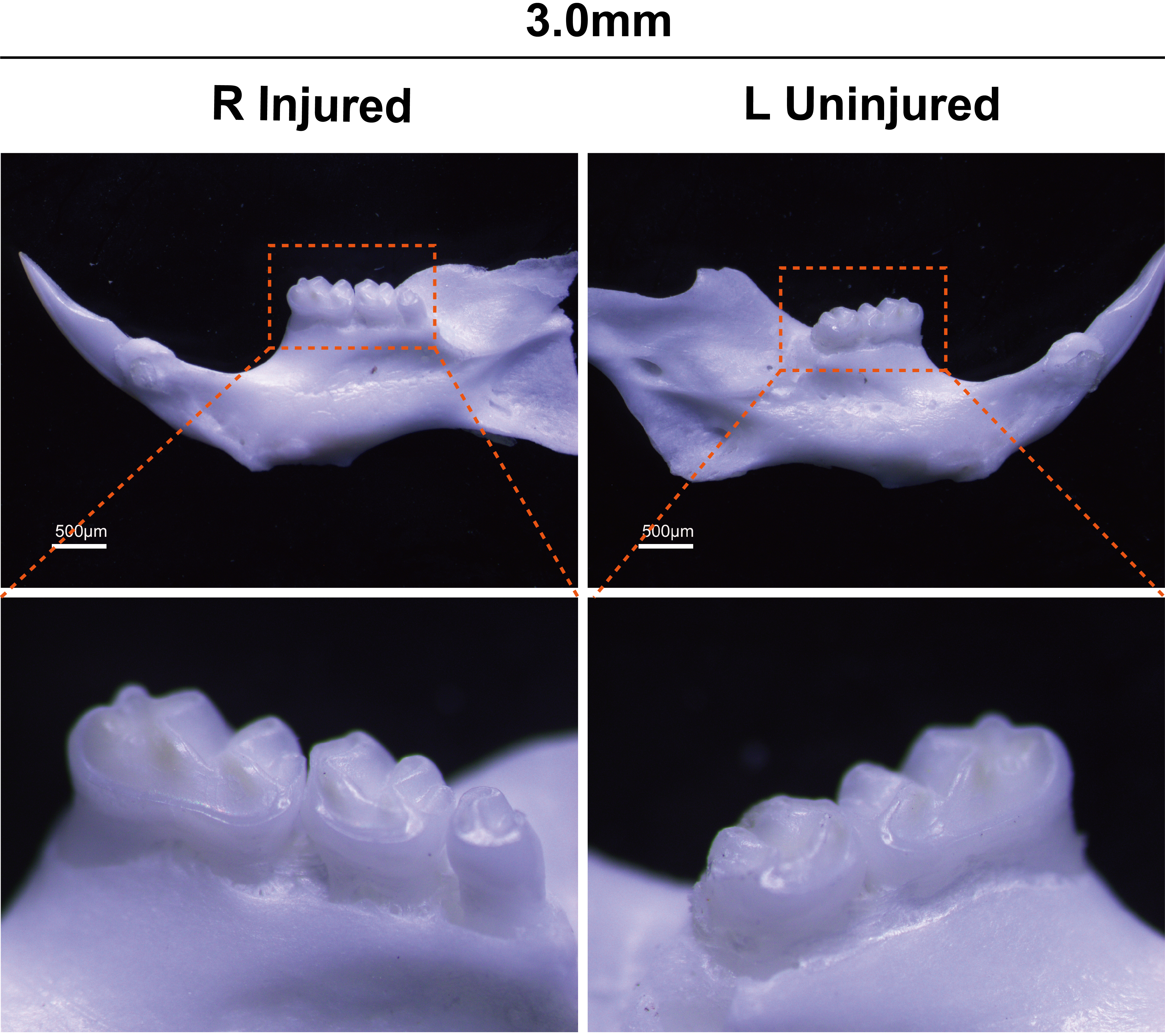

Supplement: Supplementary file 3 — Figure S3. Compare the bilateral tooth wear 28‐day recovery after unilateral VML. No significant differences between L and R tooth wear. L, left; R: right. [file CPR-57-e13610-s004.jpg]
