# Supplementary material for: A critical size volumetric muscle loss model in mouse masseter with impaired mastication on nutrition
Source: Cell Prolif. 2024 Feb 14;57(6):e13610. doi: 10.1111/cpr.13610 (PMC11150142; doi:10.1111/cpr.13610)
Supplement: Supplementary file 5 — Data S2. Supporting information. [file CPR-57-e13610-s002.docx]

**SUPPLEMENTARY MATERIALS AND METHODS**

**Tibialis anterior muscle harvest**

Mice were sacrificed 28 days after VML with an overdose of isoflurane. An incision was made to expose the tibialis anterior muscle. The ankle and knee tendons were cut with sharp scissors, and the tibialis anterior muscle was sharply separated from the surrounding tissue, and then the intact TA muscle was obtained. Sections were prepared and stained as previously described.

**Temporomandibular joints histology analysis**

After euthanasia, the mouse skulls were dissected, and samples containing temporomandibular joints tissue were fixed in 4% polyformaldehyde (Biosharp, Guangzhou, China) at room temperature for 24h followed by decalcification with 15% EDTA for 30 days. Samples were processed and embedded in paraffin, and 5-µm thick mid-sagittal sections were stained with Alcian blue and haematoxylin & eosin for morphologic analysis.

**Micro-CT scanning**

After the mice were maintained for their scheduled period, they were killed and immediately decapitated. The skull bones were harvested and fixed with 4% paraformaldehyde for 24h at room temperature. Multiple scans were then taken of the resected skull (70 kV, 114mA, slice thickness of 0.010mm) by using the micro-CT Systems (VivaCT 80, SCANCO Medical AG, Switzerland). Mimics 21.0 software was used for imaging analysis.
